# Expertise as contingency-reduction: Evidence from interviews concerning Russia’s 2022 invasion of Ukraine on German TV news

**DOI:** 10.1177/09636625261425575

**Published:** 2026-03-22

**Authors:** Monika Krause, Jan Gilles

**Affiliations:** 1 The London School of Economics and Political Science, UK

**Keywords:** expertise, nomos-building, philosophy of social science and humanities, Russian invasion of Ukraine, sociology of knowledge, TV-News, War in Ukraine

## Abstract

This article analyses interviews with experts on German television news concerning the 2022 Russian invasion of Ukraine in the context of debates about the public contribution of the social sciences and humanities. Considering the first 13 months of the war, we find that the questions put to experts are mostly not about Eastern Europe or Ukraine. Rather they concern the present and the future with reference to implications for a ‘we’ conceived as the viewing public in Germany. In their answers, academic experts do not draw on research, but they draw on academic knowledge to produce statements, which reduce the range of possible interpretations and outcomes using exclusion, scenarios and probabilities. Experts work to reduce contingency, a mode of ‘doing expertise’ that cannot be fairly characterized merely as a governance strategy and is not fully captured by debates between scientistic approaches to the social sciences and humanities and their critics.

## 1. Introduction

Scientific experts have played a prominent role in public debates on a range of national and international crises and challenges in recent decades. This includes challenges, which involve issues usually seen as owned by the natural or life sciences such as climate change, HIV-Aids, genetic engineering and Covid-19 but also issues that are more clearly claimed by scholars in the social sciences and humanities, such as terrorism, the financial crisis and the Russian War against Ukraine, which is the focus of this article.

Beginning with the buildup to the full-scale invasion on 24^th^ of February 2022, news media and talk shows created a significant demand for ‘experts’ in the US and Europe. In Germany, given both historical ties and geographical proximity, this persisted on a relatively high level for many months.

What are academics asked on these programmes? What do they say in their answers? What knowledge claims about what kind of entities are involved? This article answers these questions based on an analysis of interviews with experts on German public television news. Our material covers interviews on ‘Tagesthemen’ and ‘Heute Journal’ during the 13-months between 10^th^ February 2022 and 10^th^ March 2023, a period which includes the two weeks preceding the invasion and the two weeks following its anniversary.

Our research builds on and intervenes into previous work on experts on the news and debates about the nature of the public contribution of the social sciences and humanities. Within research on experts on the news, which we discuss in more detail further below, there has been relatively little detailed empirical attention to the objects of knowledge addressed and the knowledge claims involved, particularly within statements from scholars in the social sciences and humanities. Meanwhile, when scholars debate the nature of knowledge in the social sciences and humanities and discuss what their public role should be, they largely do so without sustained empirical attention to the statements and texts produced by their colleagues in the course of their current contributions.

Among experts interviewed on ‘Tagesthemen’ and ‘Heute Journal’, we focused specifically on contributions from academics employed by universities. These academics held positions in history, political science and economics. We analyse the questions which experts are asked, and the answers experts give with regard to what kind of entities knowledge claims are about and how they are construed; we then consider the ways experts react to explicitly normative questions and to the invitation to give recommendations for policy. For further analysis, we consider the content we observe in the context of content that could conceivably be observed in these situations but is absent.

We find that in providing expertise, academics largely do not talk about research, their own or that of others, but they draw on academic knowledge and the language created by the social sciences and humanities to produce statements which reduce the range of possible interpretations and outcomes using scenarios, exclusion and the language of probabilities. Experts neither proclaim certainty nor emphasize contingency but rather reduce contingency and make it manageable. Drawing on the sociology of knowledge, we argue that in the context of public debate in this crisis, this reduction of contingency is not adequately described as merely a strategy of governance: expertise here plays the role of a nomos-building institution (Driedger, 1980; Berger, 1967) that constructs meaningful social order and also maintains a space in which political responses can be articulated. The contributions by these experts highlight an option for ‘doing expertise’ that is not adequately theorized either by understandings of science, which see science as a journey towards the elimination of contingency, or by understandings of science, more prevalent in the humanities, which hold that it is the job of science (in the broader sense of the German ‘Wissenschaft’) precisely to highlight and produce contingency.

## 2. Literature review

The study builds on previous work in journalism and media studies, science communication and the sociology of science and the media which has examined the role of experts in the news, especially since the 1990s. The rise of the expert on TV accompanied a shift in journalistic practices, a shift away from description and a deferential stance towards government sources, towards analysis and investigative reporting (Albæk, 2011). It can also be theorized as accompanying a scientization of politics which in turn led to an increased need for the public justification of science (Bogner, 2021; Eyal, 2019; Stampnitzky, 2023; Weingart, 1983).

Previous research has examined the motivations and experiences of experts who contribute to the news (Allgaier, 2011; DiBella et al., 1991; Krüger, 1987; Peters and et al., 2008). Previous research also has highlighted the way these interactions are shaped by aspects of the production process of media organizations (Albæk, 2011; Conrad, 1999; Fenton et al., 1998; Hilgartner, 2000; Kruvand, 2012; Peters, 1994). Put briefly, journalists are looking for experts who are willing to make themselves available on short notice, answer phone calls and are not too nervous (Conrad, 1999: 290). Good sources are knowledgeable, connected to prestigious institutions, direct and articulate, willing to engage in prediction and do not overly qualify their statements.

Recently, much of the work on experts in the news has focused specifically on the ways in which experts are selected and on patterns in who gets selected. This work has explored the share of experts among sources (Cross, 2010), the institutional affiliation of featured experts (Minotakis and Tastsoglou, 2023; Morani and et al., 2022; Wagner et al., 2019), the share of academics among experts, and the correlation between appearances and measures such as citation counts (Leidecker-Sandmann and et al., 2022). Research has consistently found that women are underrepresented among sources in general and expert sources in particular (Armstrong, 2004; Crettaz Von Roten, 2011; Howell and Singer, 2017; Hubner, 2023; Joubert and et al., 2023; Kitzinger, 2008; Niemi and Pitkänen, 2017; Pallaver and Lengauer, 2008; Wagner et al., 2019).

In this field of research, the content of these media appearances and the details of what is being asked and said about what, and how, have been relatively neglected, with most exceptions focused on the natural sciences. Work which has used the analysis of media ‘frames’ (Entman, 1993; Gitlin, 1980; Goffman, 1974) for a discussions of expertise in the news, is of course relevant here (Armon and Baram-Tsabari, 2017; Da Silva Medeiros and Massarani, 2010; Laidlaw, 2019; Steele, 1995; Väliverronen, 2006; Van Der Wurff et al., 2013). But the analysis of media frames, as well as the broader analysis of how science is ‘staged’ (Hilgartner, 2000), is a technique designed precisely to move away from explicit content to include a broader set of elements of the final product circulating in the media; the analysis of media frames is also a technique deliberately geared towards rendering the content in terms of schemas and clichés.

Our focus on content and knowledge claims is also different from discourse-theoretical approaches, which tend to assimilate content produced in specific situations into more general notions of ‘political discourse’ or ‘media representations’. Discourses are often intentionally analysed without attention to speakers and their roles and with an emphasis on the relationship among elements of texts rather than specific statements. We are interested in the specific role that is created for experts intentionally by media professionals, which is negotiated by academics in variegated ways. In background interviews we conducted, TV professionals delineate the role of experts quite clearly as different from that of politicians. On air, experts are treated differently from politicians and those who are positioned as witnesses; they are, for example, not challenged in the same way.

Our analysis of knowledge claims allows us to put the text produced by these conversations in dialogue with some of the options that have been formulated for the self-understanding of scholars in the social sciences and humanities. Scholars in these fields have long debated the nature of the knowledge they produce, the ontological status of the entities they examine, as well as how they should and should not relate to the public or to different publics. These debates tend to focus on discussing the value of different explicit positionings towards ‘science’, ‘policy’ or ‘activism’, or the provision of ‘critical consciousness’ or ‘orientation’ (Bate, 2011; Burawoy, 2004; Calhoun, 2008; Messling et al., 2025; Scholtz, 1991), leaving the content of what is said in specific contexts and how it relates to different understandings of science or politics unexamined.

## 3. Data and methods

Our material covers interviews with experts on Tagesthemen and Heute Journal during the 13 months between 10^th^ February 2022 and 10^th^ March 2023, a period which includes the two weeks preceding the invasion and the two weeks following its anniversary.

The two shows are news magazines on public television, which follow up on the main news shows with more depth and reporting. Tagesthemen and Heute Journal usually last for around 35 minutes, with shorter and longer episodes in specific cases. Taken together, they are among the most-watched programmes in Germany every evening, attracting around 6.1 million viewers and a market share of 27.2% on average in 2022 (Zubayr et al., 2024: 4). The combination of reach and claim to provide background and in-depth information makes these media well-suited for our study.

In reviewing the programmes’ materials, we drew on a distinction between situational experts, such as eye-witnesses, on the one hand, and authoritative experts (Remus, 2014) on the other hand, and focused only on authoritative experts. These experts included academics, employees of think tanks and NGOs, journalists and former generals among others. We counted a total of 335 ‘authoritative’ expert appearances with a screentime of over 12 hours in the two news programmes analysed. Academics were featured in a little more than a third of the clips (37%, 124 appearances). Some of these appearances were embedded in journalistic reporting, usually lasting less than 30 seconds, with the questions often edited out (217); others were question and answer segments focused on the voice of the expert (118), lasting between about 2 and 9 minutes (see Supplemental Material Appendix 3).

Our content analysis focused on the longer interviews, specifically those conducted with academics employed by universities (37 appearances, 20 different experts). More than half of these interviews were conducted with political scientists (20), about a quarter with historians (9). The remaining interviews were conducted with economists (5), a psychologist (1), a legal scholar (1) and a neuroscientist (1). Five of 37 of these longer interviews were with female experts (13.5%).^1^ Even though interviews split almost evenly between both programmes, Tagesthemen showed a higher variety of experts than Heute Journal (16 different experts out of 20 interviews vs 6 out of 17) and a higher representation of women (5 different female experts out of 5 interviews vs 0 out of 0).

We produced verbatim transcriptions of all questions asked on both programmes. We produced verbatim transcriptions of experts’ answers on Tagesthemen. Videos for Heute Journal programmes were only accessible to the team at the ZDF office. We were not able to transcribe all statements verbatim and rely on fieldnotes we produced, which summarize each answer. Samples of both transcripts and fieldnotes are provided in Supplemental Material Appendices 1 and 2.

In approaching transcriptions and fieldnotes from this particular setting, we draw on the sociology of expertise. Despite the expression ‘scientific expertise’, the sociology of expertise starts from the assumption that expertise is neither science nor not science. Weingart (2005: 50) describes expertise as a historically evolved ‘institutional intermediate layer between science and politics’.

When scientists are on TV, certainly when they are on TV news, they are not currently ‘doing science’. The people interviewed on TV in our study are scientists (in the encompassing German sense of the term) whom we observe not doing science but ‘doing expertise’.^2^ Our questions about content are thus embedded in questions about expertise: How are scientists doing expertise? What are scientists saying when they act as experts? What knowledge claims about what kind of entities are involved?

In analysing the data, we first manually analysed the materials to list the objects of knowledge asked about in all questions in all long interviews using emergent codes (see Supplemental Material Appendix 4). We also produced lists of objects discussed in answers. We then consider the kinds of claims about objects of knowledge observed and the ways experts react to explicitly normative questions concerning different policies or courses of action. For further analysis, we consider the content we observe in the context of content that could conceivably be observed in these situations but is absent. This is part of an understanding of critique as the practice of placing an object of analysis in the context of alternative possibilities, which is different from an understanding of critique as denunciation. We include this analysis to offer further perspectives on our materials without being able to claim to be exhaustive in the alternatives we consider.

In addition to a content analysis of expert interviews on German television news (Tagesthemen and Heute Journal) concerning the Russian invasion of Ukraine between January 2021 and March 2023, we draw on supplementary data sources, which include interviews with journalists who have had involvement in producing news programmes for German public television (*n* = 2), interviews with academics in Eastern European Studies associated with universities in Germany and the UK (*n* = 40) as well as observations at conferences in those two countries and at online events during the period under study and after.

## 4. Findings

### What experts are asked

Below, we reproduce a number of the questions which experts are asked, which serve to illustrate the range and the tone of the questions in our transcriptions and field notes.

Very briefly, Professor, with your many years of experience in this area, what is your gut feeling? Will it still work, will it remain peaceful, or will there be war? (Heute Journal, Question for Neitzel, 14.02.2022, all direct quotes our translation)

But Ukraine is not Chechnya. There are a lot of cities there. Does he [Putin] want to devastate them all now because he can’t win any other way? What do you think? (Heute Journal, Question for Masala, 03.03.2022)

What is your assessment? Can Ukraine really win this war? Or is that rather to some extent wishful thinking here in the West? (Heute Journal, Question for Neitzel, 10.04.2022)

How will we all feel the effects? It’s not just about filling up the car and heating the home. (Tagesthemen, Question for Fratscher, 10.3.2022)

As these quotes illustrate, the conversations take the news as their starting point, which leads to the request to situate events in some broader classificatory context. This general type of question is sometimes asked very directly, for example in the following quote from a clip referencing a meeting between Putin and German chancellor Scholz before the Russian invasion: ‘Could you help us to assess this in its context?’ [‘Helfen Sie uns mal das Einzuordnen.’.] (Tagesthemen, Question for Sasse, 15.02.2022)

In very general terms, we would submit, journalists are asking about what is happening, about what it all means and about what will happen. Questions about meaning are sometimes about significance in the sense of ‘historical importance’. The political scientist Herfried Münkler, for example, was asked: ‘How epochal is what we are currently experiencing?’ (Tagesthemen, Question for Münckler, 11.3.2022).

We observed that the questions posed are largely not about Eastern Europe as an area or about the people affected. They are about military events and about ‘us’, the nation, the ‘we’ that is witnessing, that should act or not, that will be affected in Germany. This is very explicit in the following quote:

What do we need to be prepared for? (Tagesthemen, Question for Kooths, 28.2.2022)

Beyond significance, understood as ‘historical importance’, questions sometimes address different dimensions of meaning. Some questions could be paraphrased as: ‘Does it mean we got it all wrong?’ or ‘What can be done now?’ such as when experts are asked to assess Germany’s, or more specifically the Social Democratic Party’s, past policies towards Russia (Tagesthemen, Question for Kropp, 19.4.2022).

Although historians feature prominently, and historians are at the core of German expertise about Eastern Europe, the questions are rarely about the past but about the present and the future. The question ‘What will happen?’ is part of the question ‘What is happening and what does it all mean?’.

In public reflections on her experience of providing expertise since the beginning of the current crisis, Gwendolyn Sasse, the head of the German flagship centre for academic expertise on Eastern Europe, ZOISS, has commented on the fact that she has again and again been asked the question ‘What is Putin thinking?^3^’ Also in our corpus, this is indeed a frequently asked question. The following are both actual questions and a type that can be found adapted to a number of actual events:

What does the current course of the war tell you about Putin’s goal? (Heute Journal, Question for Masala, 25.02.2022)

Why is he doing this now? (Tagesthemen, Question for Marsala, 27.02.2022)

The question combines the news that prompts the discussion, a question about the present and a question about the future, with an attempt to access it through the entity attributed overarching causal force in the situation. Much rarer, but also asked is ‘what is [German chancellor] Scholz thinking?’ Examples of this are below.

Why doesn’t the chancellor himself communicate directly how this is supposed to work? (Tagesthemen, Question for Kropp, 15.4.2022)

Today Scholz promised money, but he continues to keep a low profile regarding the delivery of heavy weapons. Does he have no other option or doesn’t he want to? (Tagesthemen, Question for Mangott, 19.02.2022)

### Knowledge claims in experts’ answers

Academics in our sample hardly make reference to any research. In our material, the military historian Sönke Neitzel is an exception in that he references research in two examples, concerning the relative security of the cold war period and the causes of war crimes.^4^

But interviewees make knowledge claims in their responses. In reviewing the knowledge claims that academics give in response to these questions, we can note differences in what these knowledge claims are *about*. Sometimes this knowledge concerns specific factual or legal questions, such as who or what is responsible for the explosion of an under-water pipeline, whether Russia is committing genocide, or whether former chancellor’s Gerhard Schröder’s proposal for peace should be taken seriously. As they are often asked about the future, respondents also discuss claims relating to the future.

In those cases, experts largely do not claim certain knowledge, but they do reduce the range of plausible answers. Most scholars in the humanities and social sciences would argue that it is not possible to make claims about the future, though this was thought possible in some other periods in history (Loewith, 1949). In our materials, television news experts do not engage in prediction but they do reduce the contingency or openness that might be ascribed to the future.

In our transcripts, experts at times reject the demand for prediction in very explicit terms such as in the below:

Of course, I don’t have a crystal ball any more than the rest of us. Future prices of electricity are also traded on the electricity markets and you can sort of see what the experts, the companies and investors have in mind for the future and, unfortunately, things don’t look so good. In 2024 and 2025–if the markets are right – we will still have electricity prices that are several times higher than what we were used to. (Hirth, Tagesthemen 29.08.2022)

Honestly, ehm, I’m curious to see what happens. (Masala, Tagesthemen, 25.02.2022).

But experts do make claims about the future. This reduction of contingency uses a range of means. First, experts speak to questions about the future through the exclusion of certain possibilities: viewers are told not to expect Putin to give up on his territorial demands and on the demand that what would remain of Ukraine be neutralized. Viewers are told not to expect that the Ukrainian government would agree to Putin’s demands. Experts are speaking against hopes for a complete victory for Ukraine and say that it is hard to imagine that China and India would cease to support Russia.

In the contribution below, historian Sönke Neitzel also starts from the exclusion of the possibility of an overall end to the fighting, a return to ‘normalcy’. He then discusses a range of scenarios, such as a ‘kind of armistice’, a ‘frozen conflict’ (developed by analogy to the situation in Donbass) or a large offensive of Russian Army forces, which give some shape to what may or may not lay ahead.

So, in the best-case scenario, I reckon that in a few weeks there will be some kind of ceasefire – declared or not – in eastern Ukraine. But that this conflict is of course not resolved at all and that we will then have something like what we saw in the Donbass after 2014–in other words, a kind of frozen conflict that flares up from time to time. But other scenarios are also conceivable, such as the Russian armed forces launching a major offensive again after a break of a few months. So, an end to the fighting – as much as we would all like that – a return to business as usual is not in sight at all. (Neitzel, Tagesthemen 22.04.2022)

The distinction between scenarios is the basis for discussing (if not necessarily allocating) probabilities, such as in the below:

And if Ukraine continues to receive weapons, intelligence data, if it receives operational support, then Ukraine can launch further counter-offensives. However, it is more likely that we will end up in a war of attrition lasting for months, where men and material on both sides are worn out, soldiers fall, equipment is destroyed and neither side can make any significant progress on the front. Only when both sides have the impression that they can no longer achieve military success will there be a chance for possible negotiations. At the moment, however, we are still a long way from that. (Mangott, Tagesthemen, 13.12.2022)

Discussion of probability or risk make it possible to discuss outcomes that are of interest to the public, such as an escalation and expansion of the war to include NATO and possibly Germany itself:

I think these kinds of statements, these threats, we’ve been hearing them since the beginning of the war. Whenever Western nations have decided to go into the next higher category of arms deliveries, we have heard such threats from the Kremlin. So far, nothing has followed. Everything we’ve seen in terms of strategy changes from Putin in this war has centred on Ukraine and has not gone beyond Ukraine. Ultimately, I would say the risk hasn’t increased massively, but we can’t rule it out either. We simply don’t know what the point might be at which Putin would decide that this is now one point, or this is now one delivery too many, and now I’m escalating, now I’m at war with NATO. (Deitelhoff, Tagesthemen, 25.01.2023)

### Recommendations in experts’ answers

Occasionally, questions turn very explicitly to concerns about ‘what should be done’. Experts are asked in general terms about how the ‘West’, or the German government should react to a move by Putin; or they are presented with a course of action, such as sanctions against Russia or the delivery of tanks to Ukraine, and asked if that is the right path forward.

Experts largely do not avoid answering these questions. In our materials, academics go on record to say that it is necessary for Germany to decrease its dependence on Russian and Chinese energy, for Russian banks to be excluded from the international banking system, for sanctions against oligarchs to be imposed and for tanks to be delivered to Ukraine.

But, as we have discussed, the questions put to experts mostly are formulated as versions of ‘what is happening?’ or ‘what will happen’, or ‘what are the kinds of things that could be happening’, or ‘what would it be like if [something] happened?’ The specific contribution of experts as envisioned by journalists is not in the specific normative statements (which journalists routinely discuss with politicians) but in explaining and situating what is and in making more concrete what could be.

There is, however, also a recommendation underlying the questions and answers about ‘what is happening?’ and ‘what will happen?’, which is towards a specific kind of pragmatic politics. Experts rule out certain expectations, of politicians and of events, and thereby draw attention to political choices, relevant factual questions and longer-term considerations amid external constrains.

When experts rule out certain extreme possibilities, this seems to open up or help maintain a space for a specific kind of politics. Experts are repeatedly asked about the risk of nuclear escalation, for example; by discussing this risk but also putting it into the context of previous experiences with such a risk and advocating against ‘panic’, and by highlighting the need ‘to learn to live with the nuclear threat’, different options within domestic and foreign policy can be discussed.

The resulting vision of politics is made explicit in the following quote from the political scientist Nicole Deitelhoff:

That is indeed very, very difficult. Of course, we can’t let ourselves be guided by these threats. That would actually have as a consequence, that we deter ourselves from taking action in any way. That can’t be the way forward. But of course this leads to a politics that proceeds cautiously and considers every step and also examines what the consequences of these steps are and also observes what happens with certain decisions, including the supply of weapons. So actually proceeding step by step and not letting yourself drift, so to speak, and always going straight to the next stage, but instead actually seeing what happens. I think that the approach that politicians have to take here is like driving in foggy conditions where one can only see at very limited distance. But that is simply necessary in the current situation, a very dynamic conflict situation. (Deitelhoff, Tagesthemen, 25.01.2023)

Similarly, by emphasizing that the war will not be ended quickly through military means in either direction, a range of choices about the delivery or not of arms and about a range of diplomatic steps that could be taken come into view. Drawing on knowledge about past conflicts, an expert, for example, mentions prisoner exchanges and discussions about grain exports as measures that could be taken to build trust (Schröder, Heute Journal according to fieldnotes, 29.01.2023).

When concrete options are identified and considered, factual questions that arise in relation to these options come into focus. Factual questions relating to weapons delivery, for example, include questions about threat scenarios, availability and cost and the difference that different weapon systems can or cannot make.

In some cases, experts make a separation between facts on the one hand and political decisions or the ‘political will’, on the other hand, marking some element of a deference to the political sphere, such as in the below:

Well, the question is what political will exists to supply such weapons. So, what Deputy Inspector General Laubental said is not wrong. The German stock of equipment is not very large and if we were to supply heavy weapons – Marder tanks or others – the Bundeswehr would indeed lack them. But the question is: are we prepared to accept this shortage for the sake of greater security in Ukraine, because we don’t want Ukraine to lose this war. I don’t think the Russian positions are at the Seelow Heights^5^ right now, but they are in the Donetsk region and that’s where Russia must be confronted decisively, by all means, as the chancellor said. (Neitzel, Tagesthemen, 22.04.2022)

Shifting away from extreme but unlikely options also leads to the discussion of long-term issues, such as energy policy in its various dimensions and the role of critical infrastructures. An example of this is below.

Politicians and institutions must do three things in the future. Firstly, it needs to free itself much more quickly from its dependence on countries such as Russia, but also China, and especially when it comes to energy supplies to Russia, so that it is no longer susceptible to pressure, or no longer so susceptible. Secondly, of course, the expansion of renewable energies must take place much more quickly. This should have happened 10-15 years ago. So, this really needs to be prioritised. And thirdly, the third point is really about savings. We have much, much potential – in the building sector, in the transport sector – to use energy more efficiently. And these are the three measures that should have been prioritised 10-15 years ago and now it’s high time that this happened. That would also help to significantly reduce our dependence on Russian gas and oil over the coming years. But it is now really overdue to do this more quickly. (Fratscher, Tagesthemen, 10.03.2022).

### Further analysis

To gain further perspectives on the material, we try to describe what is observed in the context of what might be observed but is not. We understand critical analysis as the practice of placing an object in the context of other imaginable alternatives. A full critical analysis in this sense cannot be confined to the comparison of ‘what is’ with what has already been identified as a preferrable alternative, such as that experts should be allowed to speak longer (Verhoeven, 2010), or that they take a different policy position. We should in principle compare what we observe to *all* other versions of what might be observed, including those versions that might be considered ‘worse’ from different perspectives. These ‘worse alternatives’ are often not considered in critical analysis as a matter of course.

In our case, we can contrast the interviews with imagined television programmes where news are complemented with specific research findings to a greater extent. We can contrast the material we presented with a plausible world, in which the future is held to be predictable, either in the terms of scientific expertise or in the terms of religious or political ideology.

These conversations could be about history or Eastern Europe, or about Ukrainians directly, but they are not. They are about the situation viewed through the lens of residents of Germany and German national politics. In both questions and answers, the national framing is striking: it underlies the ‘we’ that is implied both in questions about the future and in questions about where ‘we’ have gotten it wrong, and what ‘we’ should do. This is accepted by the experts in their answers.

The relevant views are further limited to a certain political range. There are no questions, for example, about a possible contribution of NATO, or of Ukrainian policies towards minorities in these interviews. There are no questions about ‘what Zelenskyy is thinking’, which would present decisions by the Ukrainian president as a puzzle in a way that is symmetrical to the way journalists often ask about Putin. We are prompted to think that this could be otherwise by one of our background interviews with a Russian studies scholar at a university in the UK, who explained his shock at a series of ‘mad decisions’ by Zelenskyy, who, according to the respondent continued to insist on sending thousands of soldiers into certain death for the cause of Ukrainian nationalism.

The broadly pro-Ukrainian framing by the journalists is not questioned on the shows in the experts’ answers. It is hard to imagine that this relative consensus among experts is not at least partly intended by producers who select and invite the experts. According to one of our background interviews, a selection process which operates to ‘avoid’ ‘pro-Putin’ positions can be justified within public television with reference to the liberal-democratic order established by the German constitution (FdGO), which foresees freedom of opinion within a limited framework designed to protect democracy. This can be interpreted to have a foreign-political dimension. Experts are not staged here to conduct a political debate, though it has to be noted that this political debate is taking place elsewhere, including with participation of academic experts, for example in talk shows and in politics itself. Further research could examine other media as well as public meetings hosted by federal and state agencies for political education, which have emerged as prominent sites of academics’ public engagements in this conflict.

What kind of ‘we’ is imagined in these conversations on public television news? The ‘we’ is used to denote a group of people resident in Germany, who need to ‘buy gasoline’ for their cars in Germany, heat their homes, are worried about war on German territory, and seek to support Ukrainians. There is no evidence for strong assumptions about cultural unity. The conversations here lean towards advocating for military support of Ukraine, and a possible need to strengthen the German army, but there is no evidence of war mongering or expansionism. There is also no talk of past or future (German) greatness or glory and honour. We can also note that there is hardly any hostility towards Russians or Russian culture; indeed, some of the experts featured on these programmes have gone on the record to challenge claims about an association between war crimes committed by Russians and Russian culture.

## 5. Conclusion

This article has analysed interviews with experts concerning the Russian invasion of Ukraine on German television news, with a view to the objects of knowledge addressed and the knowledge claims involved. We find that experts are asked to interpret the present and speak about the future with reference to a ‘we’ that is conceived of in terms of the viewing public in Germany. In their answers, experts largely do not draw on research, their own or that of others. But they do draw on academic knowledge to produce statements, which reduce the range of possible interpretations and outcomes.

Experts use exclusion, probabilities and scenarios as techniques to reduce uncertainty about the future. These techniques have a longer history in the science and the social science and humanities where they accompany the discovery of different social forms and social states (Hacking, 1975; Lepenies, 1988; Strand and Lizardo, 2022). They also have a more recent history in the study of the future, as well as in associated techniques of governance in the Cold war era (Andersson, 2018; Dayé, 2020, 2022; Lakoff and Collier, 2010; Mallard and Lakoff, 2011).

In contrast to the ‘Cassandra phenomenon’, whereby scientists adopt a warning role in public (Weingart, 2002; Weingart et al., 2007), experts in the material we analysed adopt a role that is in some way reassuring, even if not at all optimistic. They respond to what Butler at al. have called a ‘reassurance emergency’ in technicized society (Butler et al., 2025). This ‘reassurance emergency’ can be conceived of as a specific form of anomie. Following Peter L. Berger, we could then call expertise a nomos-building institution (Driedger, 1980; Berger, 1967).

Berger’s version of anomie emphasizes vulnerability, and in particular what we might call ‘interpretative vulnerability’, the need for meaning in a shared world from a constructivist perspective (Berger, 1967:19–22). Berger and Kellner (1964: 16) discuss ‘the “existential anxiety” that probably, inevitably, accompanies the perception that nothing but one’s own narrow shoulders supports the universe in which one has chosen to live’.

The terms used in the following passage from Berger and Kellner’s classic article about marriage could apply in some ways also to the way public television provides a framework for a national processing of a sense of shock and crisis after events that led to questions about policy assumptions of the past and the future of the security of the country: ‘The plausibility and stability of the world, as socially defined, is dependent upon the strength and continuity of significant relationships in which conversation about this world can continually be carried on’ (Berger and Kellner, 1964: 4).

While much of the literature on scenarios and probability draws on Foucault to discuss how these ways of knowing are intertwined with governance and governmentality, our case is not one of governance, and our point is not mainly to note critically that these techniques reduce the range of futures that are considered. In the case of these public statements, and in view of the precarity of the shared world noted by the sociology of knowledge, a reduction of plausible options can be democratically enabling as well as constraining.

Our observations about expert interviews on TV in this case reinforce the sense that scientism and its opposite, among scientists and especially among observers of science, have hindered understanding of the actual contribution that scientists make in public. In the material we examine, experts leave a lot of room for contingency but give it shape and form and reduce rather than enlarge it. In their answers to questions on these programmes, experts chart a path between delivering predictions and emphasizing that the future is entirely unknowable.

This reduction of contingency emerges as one option for those scientists wishing to do expertise, an option that is not adequately theorized either by understandings of science, which make a strict distinction between the known and the unknown and see science as a journey towards the elimination of contingency or by understandings of science, more prevalent in the humanities, which hold that it is science’s job precisely to ‘question taken-for granted certainties’ (Rüther and Gauger, 2007) and thus to highlight contingency. The materials also challenge the opposition between scientificity and the ‘provision of orientation’ (Scholtz, 1991), which sometimes underlies discussions about the role of the humanities in different traditions. Further research can examine how warning and reassurance, as well as different forms of the ascriptions of knowability and contingency to the world are distributed across expert communities, crises and media and how they are received.

## Supplemental Material

sj-pdf-1-pus-10.1177_09636625261425575 – Supplemental material for Expertise as contingency-reduction: Evidence from interviews concerning Russia’s 2022 invasion of Ukraine on German TV news Supplemental material, sj-pdf-1-pus-10.1177_09636625261425575 for Expertise as contingency-reduction: Evidence from interviews concerning Russia’s 2022 invasion of Ukraine on German TV news by Monika Krause and Jan Gilles in Public Understanding of Science

## Notes

1. The share of women in all appearances, including very short clips in reporting segments, was higher. On Tagesthemen, the share of women among all authoritative experts on this topic was 31%, and on Heute Journal, 16%. Among university-based experts, that share was 32% and 8%, respectively.

2. In the UK context, which for better or worse has pioneered systematic, competitive and, at least in that sense critical, assessment of impact, these contributions would not count as impact according to standards as they have evolved to date because they are not linked to particular pieces of research (Audretsch et al., 2022; Franzen et al., 2016; MacDonald, 2017; Shaw et al., 2022; Watermeyer, 2016).

3. Gwendolyn Sasse, Keynote: ‘Quo Vadis Area Studies amid Russia’s war against Ukraine?’ British Association for Slavonic and East European Studies, Annual Conference, 31.03.2023.

4. See Neitzel, Heute Journal, 24.03.2022 and Neitzel, Heute Journal, 10.04.2022.

5. A town ca. 90 km east of Berlin; the site of an important Soviet offensive in 1945.
