# Supplementary material for: Expertise as contingency-reduction: Evidence from interviews concerning Russia’s 2022 invasion of Ukraine on German TV news
Source: Public Underst Sci. 2026 Mar 22;35(6):652–67. doi: 10.1177/09636625261425575 (PMC13380660; doi:10.1177/09636625261425575)
Supplement: sj-pdf-1-pus-10.1177_09636625261425575 – Supplemental material for Expertise as contingency-reduction: Evidence from interviews concerning Russia’s 2022 invasion of Ukraine on German TV news [file sj-pdf-1-pus-10.1177_09636625261425575.pdf]

Supplemental Materials

Expertise as contingency-reduction: Evidence from interviews concerning Russia's 2022 invasion of Ukraine on German TV news.

Monika Krause and Jan Gilles

Content:

Appendix 1: Sample of Transcript

Appendix 2: Sample of Fieldnotes

Appendix 3: List of Expert appearances by TV show

Appendix 4: Coding of questions Experts were asked

Appendix 1: Sample of Transcript

Tagesthemen, 24.02.2022 – Gerhard Mangott, Professor für Politikwissenschaft, Universität Innsbruck

Original (English translation below)

Q: Je länger der Krieg dauert, desto mehr Menschen dürften sich entscheiden Ihre Heimat zu verlassen. Wie lange kann dieser Krieg dauern und wie sieht die militärische Strategie Russlands aus? Das zu verstehen hilft uns jetzt Gerhard Mangott, Professor für Politikwissenschaft and der Universität Innsbruck. Guten Abend.

Q: Wir haben es gehört von unserem Korrespondenten Angriffe in allen Teilen des Landes. Wir müssen davon ausgehen, dass Putin die gesamte Ukraine besetzen will.

- *Ja. Er wird vielleicht der Ukraine ihre Eigenstaatlichkeit nehmen. Vielleicht begnügt er sich damit ihre Souveränität zu beschränken, ihre außenpolitische Handlungsfreiheit. Er hat ja ganz klar formuliert, was seine politischen Ziele sind. Die Neutralisierung der Ukraine, die Entwaffnung der Ukraine. Und dass die derzeitige oder jede zukünftige Regierung der Ukraine die Krim als Teil Russlands anerkennt. Putin will die politische und militärische Kapitulation der Ukraine und er ist bereit jedes Mittel, aber wirklich jedes Mittel einzusetzen, um dieses Ziel zu erreichen.*

Q: In welcher Phase eines Angriffs befinden wir uns jetzt? Wie müssen wir uns das vorstellen, weil wir so wenig Überblick haben? Rechnen Sie noch mit großflächigem Einsatz von Bodentruppen?

- *Ja damit ist zu rechnen. Wir befinden uns eigentlich erst in der Anfangsphase dieser militärischen Aggression durch Russland. Das wird weiter gehen, das wird sich intensivieren, solange bis die geopolitischen Ziele für Putin, für Russland erfüllt sind*

Q: Kann die ukrainische Arme diesen Angriffen überhaupt Stand halten?

- *Nein. Sie ist qualitative schwächer als die russischen Streitkräfte. Sie hat vor allem Probleme bei der Luftwaffe, in der Luftabwehr und bei der Kriegsmarine. Sie kann in diesen Bereichen den russischen Streitkräften nichts entgegensetzen. Die Streitkräfte der Ukraine werden Widerstand leisten, aber sie werden diesen Krieg letztlich verlieren. Die Frage ist: welchen Blutzoll wird das erfordern, auf ukrainischer Seite, aber eben auch auf russischer Seite. Wie viele russische Soldaten werden in Särgen nach Russland zurückkehren und wie wird das die russische Bevölkerung aufnehmen?*

Q: Genau das ist es ja was Putin immer vermeiden wollte, dass es viele Opfer auch auf der russischen Seite gibt. Wäre er wohl bereit diesen Preis diesmal zu zahlen?

- *Bei all den militärischen Aggressionen Russlands in der Vergangenheit – bei Georgien 2008, 2014 auf der Krim, 2015 und seitdem in Syrien – war Putin immer darauf bedacht, dass möglichst wenige russische Soldaten sterben. Er wusste, dass das an der Heimatfront zur Gegenwehr, zu Widerspruch führt. Jetzt ist aber ganz offensichtlich bereit solche Opferzahlen hinzunehmen, weil er glaubt, dass er nach Innen alles unter Kontrolle hat. Wir dürfen nicht vergessen, in den letzten zwei Jahren gab es eine repressive Welle gegen die Opposition. Oppositionelle wurden Inhaftiert, vergiftet, ins Exil getrieben, oppositionelle Medien wurden ausgeschaltet, NGOs wurden ausgeschaltet. Er hat also schon in Vorbereitung auf diese Handlung heute, jeden Quell eines Protestes gegen sein außenpolitisches Abenteuertum in Russland erstickt.*

Q: Könnte diesem Land überhaupt noch militärisch geholfen werden? Die Ukraine fordert ja auch heute weiter wieder vom Westen mehr Waffen.

- *Nun man kann Waffen liefern natürlich. Man kann auch ökonomische Stabilisierungshilfe leisten. Aber der Ukraine wirklich helfen auf dem Schlachtfeld würde ein Eingreifen auf militärischer Ebene von den NATO-Staaten. Das wurde von*

vornherein ausgeschlossen, zu Recht. Denn das würde das Risiko einer nuklearen Eskalation nach sich ziehen, eine Eskalation die Putin heute verklausuliert auch angedroht hat.

Q: Hätte es denn überhaupt noch einen Weg gegeben diesen Krieg zu verhindern?

- *Vielleicht. Vielleicht wurden nicht alle diplomatischen Möglichkeiten ausgenutzt. Klar ist: Russland ist der Aggressor, der Angriff ist durch nichts zu rechtfertigen und es gibt eine ausschließliche Verantwortung Russlands dafür. Aber die Frage ist, ob man sich in den Verhandlungen zuvor nicht in die Niederungen realer Machtpolitik hätte begeben können und verantwortungsethisch umgegangen wäre. Denn wenn die USA davon ausgegangen sind, dass Putin die Ukraine angreifen wird, wenn die USA erklärt haben, dass sie der Ukraine militärisch nicht zur Hilfe kommen werden, und wenn klar war, das die ukrainischen Streitkräfte letztlich verlieren werden, warum hat man sich nicht durchgerungen einen Kompromiss über die Neutralisierung der Ukraine zu finden? Ich weiß nicht, ob Putin das akzeptiert hätte. Vielleicht hat Putin auch nie aufrecht formuliert. Aber was wäre verantwortungsethisch gedacht, für das ukrainische Volk besser? Eine aufgezwungene Neutralität, oder dieser verheerende Angriffskrieg durch Russland?*

Q: Aber das hätte ja vor allem die Ukraine nicht gewollt.

- *Die Ukraine hat das klar abgelehnt. Die politische Führung hat es abgelehnt. Hätte Selenskyj das auch nur angedacht, er wäre unter Druck gekommen, er hätte vielleicht sogar seine Präsidentschaft verloren, denn die Nato-Mitgliedschaft ist Verfassungsziel. Darüber besteht – mit Ausnahme der russlandfreundlichen Opposition – Konsens in der Ukraine. Es hätte also eine aufgezwungene Neutralität sein müssen. Das ist gegen unsere Werte, das ist gegen die Sittlichkeit, aber vielleicht wäre es ein nüchterner verantwortungsethischer Ausweg gewesen. Vielleicht. Wir wissen es nicht*

Q: Putin will die Sicherheit in Europa neu ordnen, so sagt er das selbst. Was glauben Sie wie weit sein Plan geht? Gehören dazu auch die Nato-Verbündeten Polen, Lettland, Estland Litauen?

- *Nun, Putin ist zur äußersten Gewalt bereit, aber ich glaube nicht, dass er es so weit treiben würde NATO-Mitgliedsstaaten anzugreifen, die unter der Bündnisgarantie des Artikel 5 stehen. Das ist etwas, was Russland nicht will. Auch das würde von beiden Seiten die Gefahr einer nuklearen Eskalation beinhalten. Und die wollen beide Seiten nicht, der Westen nicht und Russland nicht. Aber es wird notwendig sein, in den kommenden Monaten und Jahren, die Militärpräsenz der NATO in diesen osteuropäischen Staaten zu verstärken. Also die NATO-Russland Grundakte von 1998 hinter sich zu lassen, wo die NATO versprochen hat, keine substanziellen Truppen grundsätzlich permanent in den osteuropäischen Mitgliedsstaaten zu stationieren. Das ist nun Geschichte. Diese Vereinbarung hat Russland heute zerstört. Jetzt muss diese Antwort einer militärischen Hochrüstung und eine Konzentration westlicher NATO-Soldaten an den Ostgrenzen des Bündnisses geben.*

Q: Der Appell des Politikwissenschaftlers Gerhard Mangott. Danke für das Gespräch.

English Translation

Q: The longer the war is going on for, the more people might decide to leave their homes. How long can this war last and what is Russia's military strategy? – to understand these questions we are now talking to Gerhard Mangott, Professor of Political Science at Universität of Innsbruck. Good evening!

- *Good evening!*

Q: As we just heard from our correspondent, there are attacks in all part of the country. We have to assume that Putin wants to occupy all of Ukraine.

- *Yes. Maybe he will take Ukraine's status as a state away. Maybe he settles with limiting Ukraine's sovereignty, it's liberty to engage in foreign policy. He has very clearly articulated what is political goals are. The neutralisation of Ukraine, the disarmament of Ukraine. And that the current or any future government of Ukraine recognized Crimea as part of Russia. Putin wants the political and the military capitulation of Ukraine and he is ready to use any mean, really any mean, to reach this goal.*

Q: In what phase of the invasion are we currently? How do we have to understand this, as we have such little overview of the situation? Are you expecting a large-scale use of soldiers on the ground?

- *Yes, that's to expect. We are actually only at the beginning of this military Aggression by Russia. It will continue, it will intensify, until Putin, until Russia reached its geopolitical goals.*

Q: Can Ukraine actually resist these attacks?

- *No. Ukraine is qualitatively weaker than the Russian forces. Particularly it has problems in the air force, the air defence and the marine. In these fields, Ukraine has little to oppose the Russian forces with. The Ukrainian forces will resist, but in the long run they will lose this war. The question is: How much blood will that demand, both on the Ukrainian and the Russian side. How many Russian soldiers will return to Russia in a coffin and how will the Russian population take this up?*

Q: Putin always wanted to avoid that there are many casualties on the Russian side. This time, would he be ready to pay this price?

- *In all of Russia's military aggressions in the past – in Georgia in 2008, in Crimea in 2014, in Syria in 2015 and since then – Putin has always been careful to ensure that as few Russian soldiers as possible die. He knew that this would lead to resistance and opposition on the home front. Now, however, he is clearly prepared to accept such casualties because he believes he has everything under control at home. We must not forget that there has been a wave of repression against the opposition over the last two years. Opposition figures have been imprisoned, poisoned, driven into exile, opposition media outlets have been shut down, NGOs have been shut down. So, in preparation for today's action, he has already stifled any source of protest against his foreign policy adventurism in Russia.*

Q: Is there actually still a way to help Ukraine on a military level? Also, today there were calls for support with weapons from the West?

-
- *Well, of course, weapons can be supplied. Economic stabilisation aid can also be provided. But what would really help Ukraine on the battlefield would be military intervention by NATO countries. This was ruled out from the outset, and rightly so. Because that would entail the risk of nuclear escalation, an escalation that Putin has also implicitly threatened today.*

Q: Would there even have been a way to avoid this war?

- *Perhaps. Perhaps not all diplomatic options were exhausted. One thing is clear: Russia is the aggressor, the attack cannot be justified by anything, and Russia bears sole responsibility for it. But the question is whether it would have been possible to avoid descending into the depths of real power politics in the negotiations beforehand and to have acted in an ethically responsible manner. After all, if the US assumed that Putin would attack Ukraine, if the US declared that it would not come to Ukraine's aid militarily, and if it was clear that the Ukrainian armed forces would ultimately lose, why was no compromise reached on the neutralisation of Ukraine? I do not know whether Putin would have accepted this. Perhaps Putin never made a formal proposal. But what would have been better for the Ukrainian people from an ethical point of view? Imposed neutrality, or this devastating war of aggression by Russia?*

Q: But that was something that Ukraine didn't want.

- *Ukraine has clearly rejected this. The political leadership has rejected it. If Zelensky had even considered it, he would have come under pressure and might even have lost his presidency, because NATO membership is a constitutional goal. There is consensus on this in Ukraine, with the exception of the pro-Russian opposition. So, it would have had to be enforced neutrality. That is against our values, it is against morality, but perhaps it would have been a sober, responsible ethical way out. Perhaps. We do not know.*

Q: Putin wants to reorder security in Europe, that is what he says himself. What do you think how far his plan goes? Does it include the NATU-Members Polen, Latvia, Estonia, Lithuania?

- *Well, Putin is prepared to use extreme force, but I don't think he would go so far as to attack NATO member states that are covered by the alliance guarantee under Article 5. That is something Russia does not want. It would also entail the risk of nuclear escalation on both sides. And neither side wants that, neither the West nor Russia. But it will be necessary to strengthen NATO's military presence in these Eastern European states in the coming months and years. This means leaving behind the 1998 NATO-Russia Founding Act, in which NATO promised not to station any substantial troops permanently in Eastern European member states. That is now history. Russia has destroyed that agreement today. Now there must be a response in the form of military rearmament and a concentration of Western NATO soldiers on the alliance's eastern borders.*

Q: The appeal of the political scientist Gerhard Mangott. Thank you

Appendix 2: Sample of Fieldnotes

Heute Journal, 14.02.2022 – Sönke Neitzel, Prof. für Geschichte, Universität Potsdam

Original (English translation below)

Einführung: Professor Sönke Neitzel von der Uni Potsdam ist der derzeit einzige Professor für Militärgeschichte in Deutschland. Herr Professor, schön, dass Sie bei uns sind. Guten Abend!

Q: Kann es Lehren aus der Geschichte geben, wie wir die aktuelle Lage in den Griff bekommen?

- *Geschichte Internationaler Konflikte offenbart Logiken aber keine Eins zu Eins Übertragung.*
- *Müssen letztlich diese Konflikte selber lösen.*

Q: Nehmen wir an das der russische Präsident eine Kosten-Nutzen-Abwägung. Was spricht militärisch aus Putins Sicht für eine Invasion und was dagegen?

- *Dafür: Nato wird nicht eingreifen. Er kann es machen. Russland ist militärisch Überlegen. Keiner bezweifelt das sie Kiew oder den Bereich östlich des Dnepr besetzen könnten*
- *Dagegen: Haben Überraschung nicht auf ihrer Seite. Mir erscheint es eher unwahrscheinlich. Ist aber nur unser eigenes Rational und nicht das von Putin.*

Q: Dann gucken wir doch mal – weil wir nicht in Putins Kopf reinschauen können – auf das was unbestreitbar ganz offensichtlich auf dem Tisch liegt: über 100.000 Soldaten sind da aufmarschiert. Selbst für jemanden wie den russischen Präsidenten eine gewaltige Zahl. Das sind auch gewaltige Kosten für sein Land. Wie kommt er da wieder gesichtswahrend raus?

- *Kostenfaktor für ihn nicht so entscheidend. Muss Bevölkerung und Machtapparat einen Erfolg vorweisen.*
- *Alle sind ratlos, weil er sehr hoch gepokert hat.*

Q: Alle ratlos haben Sie gesagt. Bundeskanzler muss jetzt aber einen Plan haben in irgendeiner Form. Er fährt morgen nach Moskau. Hat heute in Kiew gesagt „Wir sind bereit für einen Sicherheitsdialog. Sie haben die Forderungen des Kremls gerade genannt, die sind für die Nato komplett inakzeptabel, weil sie gesamte Geschichte Europas um dreißig Jahre zurückdrehen würden. Wie soll denn so ein Sicherheitsdialog dann aussehen?

- *Einzige Möglichkeit: die Finnlandisierung der Ukraine. Kein Nato-Eintritt aber gesellschaftliche Orientierung Richtung Westen.*
- *Aber Scholz kann nicht für Ukraine sprechen.*
- *Aber Nato und Westliche Alliierte werden kein Schriftstück unterzeichnen in dem steht das die Ukraine niemals Mitglied der Nato wird.*

Q: Gibt Menschen, die sagen schon hier darüber zu reden, was wir Putin anbieten könnten, bedeutet in seine Falle getappt zu sein. Erinnere mich da an ein Gespräch von vor ein paar Tagen hier im Heute Journal mit der estnischen Ministerpräsidentin geführt habe. Die hat genau das gesagt. Wie sehen Sie das?

- *Verständlich aus estnischer Sicht. Aber historisch gesehen kann ich sagen: Diplomatie spielt eine Rolle. In allen historischen Krisen war es vorher nicht klar wie es ausgehen würde und am Ende spielte Diplomatie immer eine Rolle.*
- *Töricht wenn Westen sich nur auf militärische Abschreckung konzentriert.*
- *Diplomatische Verhandlungen sind notwendig, neben anderen Faktoren.*

Q: Mit Ihrer langjährigen Erfahrung in dem Gebiet: Was haben Sie für ein Gefühl, Bauchgefühl? Klappt das noch, bleibt es friedlich, oder gibt es Krieg?

- *Hochrangige Mitarbeiter der Bundeswehr waren relativ cool, daher glaubt er nicht, das es Mittwoch zu einem Krieg kommen wird*

Q: Sagt Professor Sönke Neitzel von der Universität Potsdam. Vielen Dank für das Gespräch.

English Translation:

Q: Professor Sönke Neitzel from the University of Potsdam is currently the only professor of military history in Germany. Professor, it's great to have you here. Good evening!
Can we learn any lessons from history that can help us to deal with the current situation?

- *The history of international conflicts reveals certain patterns, but these cannot be applied one-to-one.*
- *Ultimately, we have to resolve these conflicts ourselves.*

Q: Let us assume that the Russian president is weighing up the costs and benefits. From Putin's point of view, what are the military reasons for and against an invasion?

- *In favour: NATO will not intervene. He can do it. Russia is militarily superior. No one doubts that they could occupy Kyiv or the area east of the Dnieper.*
- *Against: They do not have the element of surprise on their side. It seems rather unlikely to me. But that is only our own reasoning, not Putin's.*

Q: Since we cannot read Putin's mind, let us look at what is undeniably obvious: over 100,000 soldiers have been deployed there. Even for someone like the Russian president, that is a huge number. It also represents a huge cost for his country. How can he save his image?

- *The cost factor is not so decisive for him. He has to show the population and the power apparatus that he has been successful.*
- *Everyone is clueless, because he has raised the stakes so high.*

Q: You said everyone is clueless. But the Chancellor must now have some kind of plan. He is travelling to Moscow tomorrow. Today in Kyiv, he said, 'We are ready for a dialogue about security.' You have just mentioned the Kremlin's demands, which are completely unacceptable to NATO because they would turn back the entire history of Europe by thirty years. What form would such a security dialogue take?

- *The only possibility is the Finlandization of Ukraine. No NATO membership, but a social orientation towards the West.*
- *But Scholz cannot speak for Ukraine.*
- *However, NATO and the Western allies will not sign any document stating that Ukraine will never become a member of NATO.*

Q: There are people who say that even talking about what we could offer Putin here means falling into his trap. Reminds me of a conversation I had a few days ago here on Heute Journal with the Estonian Prime Minister. She said exactly that. What is your view?

- *It's understandable from Estonia's point of view. But historically speaking, I can say that diplomacy plays a role. In all historical crises, it was not clear beforehand how they would end, and in the end, diplomacy always played a role.*
- *It would be foolish for the West to focus solely on military deterrence.*
- *Diplomatic negotiations are necessary, among other factors.*

Q: With your long-term experience in the field, what is your gut feeling? Will it still work out, will it remain peaceful, or will there be war?

- *High-ranking members of the German Armed Forces were relatively calm, so he does not believe that war will break out on Wednesday.*

Q: Says Professor Sönke Neitzel from the University of Potsdam. Thank you very much for talking to us.

Appendix 3: List of Expert appearances by TV show

(see next page)

HEUTE JOURNAL

NO	DATE	LENGTH	NAME	CATEGORY	DISCIPLINE	AFFILIATION	GENDER
1	10.02.2022	18	Karbalewitsch, Walerij	Ukraine	Political scientist	Freelance; Expert on Belarus	Male
2	12.02.2022	22	Drescher, Jörg	NGO	NGO	Deutsch-Ukrainisches Forum e.V.	Male
3	14.02.2022	385	Neitzel, Sönke	Academia	Historian	Universität Potsdam	Male
4	15.02.2022	338	Heusgen, Christoph	International	International	Munich Security Conference, Chairman	Male
5	15.02.2022	40	Federico, Tobias	Consultancy	Consultancy	Energy Brainpool	Male
6	15.02.2022	42	Pepe, Jacobo Maria	Think Tank	Political scientist	SWP (Stiftung Wissenschaft und Politik)	Male
7	17.02.2022	16	Grozev, Christo	Journalism	Journalism	Research Collective Bellingcat	Male
8	18.02.2022	12	Stelzenmüller, Constanze	Think Tank	Political scientist	Think tank Brookings Institution	Female
9	20.02.2022	338	Fischer, Sabine	Think Tank	Political scientist	SWP (Stiftung Wissenschaft und Politik)	Female
10	21.02.2022	296	Masala, Carlo	Academia	Political scientist	Bundeswehr Universität München	Male
11	22.02.2022	30	Kamin, Katrin	Academia	Economist	Kiel Institut für Weltwirtschaft	Female
12	22.02.2022	58	Schulze Wessel, Martin	Academia	Historian	LMU München	Male
13	24.02.2022	219	Ramms, Egon	Military	Military	NATO, Ex-General	Male
14	24.02.2022	238	Meister, Stefan	Think Tank	Political scientist	Deutsche Gesellschaft für Auswertige Politik	Male
15	25.02.2022	261	Masala, Carlo	Academia	Political scientist	Bundeswehr Universität München	Male
16	25.02.2022	295	Feldmayr, Gabriel	Academia	Economist	Österreichisches Institute für Wirtschaftsforschung, Direktor	Male
17	25.02.2022	20	Jakobowski, Jakub	Think Tank	Political Scientist	Centre for Eastern Studies, Warsaw	Male
18	25.02.2022	25	Zhao, Tong	Think Tank	Political scientist	Carnegie Foundation for International Peace China	Male
19	26.02.2022	186	Ramms, Egon	Military	Military	NATO, Ex-General	Male
20	28.02.2022	54	Cirincione, Joseph	Think Tank	Political scientist	Quincy Institute for Responsible Statecraft	Male
21	28.02.2022	363	Zorn, Eberhard	Military	Military	Bundeswehr, Generalinspekteur	Male
22	28.02.2022	20	Kater, Ulrich	Industry	Economist	DeKaBank, Chief economist	Male
23	01.03.2022	446	Vad, Erich	Military	Military	Bundeswehr, Ex-General	Male
24	01.03.2022	436	Chodorkowski, Michail	Industry	Industry	Businessman; Regime Critic	Male
25	03.03.2022	9	Umbach, Frank	Academia	Political scientist	Universität Bonn	Male
26	03.03.2022	281	Masala, Carlo	Academia	Political scientist	Bundeswehr Universität München	Male
27	04.03.2022	24	Smital, Heinz	NGO	Physicist	Greenpeace	Male
28	05.0.3.2022	326	Semenyi, Oleksiy	Think Tank	Political scientist	Institute for Global Transformation, Kiew	Male
29	05.03.2022	324	Knaus, Gerald	Think Tank	Political scientist	Think tank Europäische Stabilitionsinitiative	Male
30	05.03.2022	27	Chieh-Chang Huang, Alexander	Academia	Political scientist	University Taiwan	Male
31	05.03.2022	29	Heilmann, Sebastian	Academia	Political scientist	Universität Trier, Professor	Male
32	07.03.2022	258	Awdeweja, Maria	Ukraine	Political scientist		Female
33	07.03.2022	17	Federico, Tobias	Consultancy	Consultancy	Energy Brainpool, CEO	Male
34	07.03.2022	14	Zachmann, Georg	Think Tank	Political scientist	Think tank Bruegel	Male
35	08.03.2022	44	Jeßberger, Florian	Academia	Legal	Humboldt Universität; Professor	Male
36	08.03.2022	31	Ischinger, Wolfgang	International	International	Munich Security Conference, Ex-Chairman	Male
37	08.03.2022	18	Füchs, Ralf	Think Tank	Politician	Think tank Zentrum Liberale Moderne	Male
38	09.03.2022	23	Grimm, Veronika	Academia	Economist	Univerisität Erlangen-Nürnberg	Female
39	09.03.2022	24	Eckart, Kai	Journalism	Journalism	Energie Informationsdienst, Editor in Chief	Male
40	09.03.2022	21	Halver, Robert	Industry	Industry	Baader Bank, Analyst	Male
41	10.03.2022	322	von Fritsch, Rüdiger	Politics	Politician	Ex-German Ambassador in Russia	Male
42	11.03.2022	304	Domröse, Hans-Lothar	Military	Military	Bundeswehr, Ex-General	Male
43	17.03.2022	25	Kaim, Markus	Think Tank	Political scientist	SWP (Stiftung Wissenschaft und Politik)	Male
44	18.03.2022	330	Awdeewa, Maria	Ukraine	Political scientist		Female
45	20.03.2022	40	Subow, Andrej	Ukraine	Political scientist	Freelance; Regime critic Russia	Male
46	20.03.2022	390	Sindeewa, Natalja	Journalism	Journalism	Broadcast station Doschd	Female
47	22.03.2022	62	Münkler, Herfried	Academia	Political scientist	Humboldt Universität	Male
48	22.03.2022	36	Neitzel, Sönke	Academia	Historian	Universität Potsdam, Professor	Male
49	24.03.2022	318	Neitzel, Sönke	Academia	Historian	Universität Potsdam, Professor	Male
50	27.03.2022	197	Kaim, Markus	Think Tank	Political scientist	SWP (Stiftung Wissenschaft und Politik)	Male
51	28.03.2022	27	Südekum, Jens	Academia	Economist	Universität Düsseldorf, Professor	Male
52	29.03.2022	369	Masala, Carlo	Academia	Political scientist	Bundeswehr Universität München	Male
53	01.04.2022	15	Weidenfeld, Jan	Think Tank	Political scientist	Mercator Institute for China Studies	Male
54	04.04.2022	283	Michalski, Wenzel	NGO	NGO	Human Rights Watch Germany	Male
55	04.04.2022	23	Südekum, Jens	Academia	Economist	Universität Düsseldorf, Professor	Male
56	06.04.2022	19	Füchs, Ralf	Think Tank	Politician	Think tank Zentrum Liberale Moderne	Male
57	10.04.2022	17	Rackwitz, Klaus	Consultancy	Legal	International Academy Nurnberg Principles, Director	Male
58	10.04.2022	47	Jeßberger, Florian	Academia	Legal	Humboldt Universität	Male
59	10.04.2022	305	Neitzel, Sönke	Academia	Historian	Universität Potsdam, Professor	Male
60	11.04.2022	28	Masala, Carlo	Academia	Political scientist	Bundeswehr Universität München	Male
61	11.04.2022	37	Mölling, Christian	Think Tank	Political scientist	Deutsche Gesellschaft für Auswertige Politik	Male
62	11.04.2022	15	Bendel, Petra	Academia	Political scientist	Universität Erlangen-Nürnberg;	Female
63	13.04.2022	23	Meister, Stefan	Think Tank	Political scientist	Deutsche Gesellschaft für Auswertige Politik	Male
64	13.04.2022	31	Füchs, Ralf	Think Tank	Politician	Think tank Zentrum Liberale Moderne	Male
65	16.04.2022	17	Kaim, Markus	Think Tank	Political scientist	SWP (Stiftung Wissenschaft und Politik)	Male
66	19.04.2022	308	Ramms, Egon	Military	Military	NATO, Ex-General	Male
67	25.04.2022	15	Ajaka, Jassem	Academia	Economist	Lebanese University Beirut	Male
68	25.04.2022	202	Frick, Martin	International	International	World Food Programme, Director Germany	Male
69	04.05.2022	304	Neitzel, Sönke	Academia	Historian	Universität Potsdam, Professor	Male
70	09.05.2022	301	Ramms, Egon	Military	Military	NATO, Ex-General	Male
71	10.05.2022	148	Snyder, Timothy	Academia	Historian	Yale University	Male
72	17.05.2022	341	Ramms, Egon	Military	Military	NATO, Ex-General	Male
73	20.05.2022	18	Politi, Marco	Journalism	Journalism	Freelance	Male
74	25.05.2022	276	Gressel, Gustav	Think Tank	Political scientist	European Council on Foreign Relations	Male
75	31.05.2022	30	Zachmann, Georg	Think Tank	Political scientist	Think tank Bruegel	Male
76	03.06.2022	375	Zick, Andreas	Academia	Psychologist	Universität Bielefeld, Professor	Male
77	15.06.2022	372	Masala, Carlo	Academia	Political scientist	Bundeswehr Universität München	Male
78	27.06.2022	360	Gressel, Gustav	Think Tank	Political scientist	European Council on Foreign Relations	Male
79	30.06.2022	351	Heusgen, Christoph	International	International	Munich Security Conference, Chairman	Male
80	05.07.2022	19	Rossolinski-Liebe, Grzegorz	Academia	Historian	FU Berlin	Male
81	05.07.2022	41	Hrytsak, Jaroslaw	Academia	Historian	Ukrainian Catholic University	Male
82	20.07.2022	12	Zachmann, Georg	Think Tank	Political scientist	Think tank Bruegel	Male
83	03.08.2022	352	Neitzel, Sönke	Academia	Historian	Universität Potsdam, Professor	Male
84	04.08.2022	45	Grossi, Rafael	International	International	IAEA, Director	Male
85	04.08.2022	31	Smital, Heinz	NGO	Physicist	Greenpeace	Male
86	06.08.2022	15	Gressel, Gustav	Think Tank	Political scientist	European Council on Foreign Relations	Male
87	11.08.2022	20	Stransky, Sebastian	Consultancy	Consultancy	Gesellschaft für Anlagen- und Reaktorsicherheit	Male
88	11.08.2022	354	Gressel, Gustav	Think Tank	Political scientist	European Council on Foreign Relations	Male
89	18.08.2022	12	Shelest, Hanna	Think Tank	Political scientist	Think Tank Ukrainian Prism	Female
90	23.08.2022	15	Trebesch, Christoph	Academia	Economist	Kiel Institut für Weltwirtschaft	Male
91	31.08.2022	19	Kosharna, Olga	Consultancy	Consultancy	Independent Expert for Reactor security	Female

92	07.09.2022	47	Masala, Carlo	Academia	Political scientist	Bundeswehr Universität München	Male
93	09.09.2022	29	Masala, Carlo	Academia	Political scientist	Bundeswehr Universität München	Male
94	11.09.2022	218	Ramms, Egon	Military	Military	NATO, Ex-General	Male
95	15.09.2022	19	Ischinger, Wolfgang	International	International	Munich Security Conference, Ex-Chairman	Male
96	15.09.2022	11	Hodges, Ben	Military	Military	Ex-Commander of US Troups in Europe	Male
97	20.09.2022	353	Fischer, Sabine	Think Tank	Political scientist	SWP (Stiftung Wissenschaft und Politik)	Female
98	21.09.2022	16	Behrends, Jan	Academia	Historian	Europa Univerisität Viadrina Frankfurt	Male
99	25.09.2022	15	Rogow, Kirill	Think Tank	Political scientist	Gaidar Institute for Economic Policy	Male
100	25.09.2022	335	Major, Claudia	Think Tank	Political scientist	SWP (Stiftung Wissenschaft und Politik)	Female
101	29.09.2022	15	Pothier, Fabrice	Military	Military	NATO, Ex-Strategist	Male
102	29.09.2022	16	Kühn, Ulrich	Academia	Political scientist	Institute für Friendsforschung und Sicherheitspolitik, Hamburg	Male
103	30.09.2022	225	Masala, Carlo	Academia	Political scientist	Bundeswehr Universität München	Male
104	04.10.2022	73	Grossi, Rafael	International	International	IAEA, Director	Male
105	08.10.2022	18	Major, Claudia	Think Tank	Political scientist	SWP (Stiftung Wissenschaft und Politik)	Female
106	11.10.2022	20	Mangott, Gerhard	Academia	Political scientist	Univerisität Innsbruck, Professor	Male
107	11.10.2022	16	Petraeus, David	Military	Military	CIA, Ex-Director	Male
108	17.10.2022	245	Franke, Ulrike	Think Tank	Political scientist	European Council on Foreign Relations	Female
109	26.10.2022	12	Snyder, Timothy	Academia	Historian	Yale University	Male
110	26.10.2022	16	Hodges, Ben	Military	Military	Ex-Commander of US Troups in Europe	Male
111	09.11.2022	16	Keupp, Markus	Academia	Economist	ETH Zürich, Militärakademie	Male
112	09.11.2022	184	Stelzenmüller, Constanze	Think Tank	Political scientist	Think tank Brookings Institution	Female
113	11.11.2022	215	Major, Claudia	Think Tank	Political scientist	SWP (Stiftung Wissenschaft und Politik)	Female
114	20.11.2022	20	Högbladh, Stina	Academia	Political scientist	University of Uppsala, Institute für Peace and conflict studies	Female
115	20.11.2022	17	Hasenclever, Andreas	Academia	Political scientist	Universität Tübingen; Professor	Male
116	25.11.2022	16	Masala, Carlo	Academia	Political scientist	Bundeswehr Universität München	Male
117	22.12.2022	228	Wiegold, Thomas	Journalism	Journalism	Freelance	Male
118	27.12.2022	18	Mölling, Christian	Think Tank	Political scientist	Deutsche Gesellschaft für Auswertige Politik	Male
119	05.01.2023	370	Masala, Carlo	Academia	Political scientist	Bundeswehr Universität München	Male
120	27.01.2023	17	Zick, Andreas	Academia	Political scientist	Universität Bielefeld, Professor	Male
121	27.01.2023	21	Sauer, Frank	Academia	Political scientist	Bundeswehr Universität München	Male
122	27.01.2023	16	Richter, Wolfgang	Military	Military	Oberst a. D.	Male
123	27.01.2023	14	Arnold, Ed	Think Tank	Military	Royal United Service Institute London	Male
124	29.01.2023	304	Schröder, Hans-Henning	Academia	Political scientist	Universität Wismar, Ostinstitut	Male
125	30.01.2023	16	Neitzel, Sönke	Academia	Historian	Universität Potsdam, Professor	Male
126	02.02.2023	41	Gloger, Katja	Journalism	Journalism	Stern	Female
127	02.02.2023	46	Sapper, Manfred	Journalism	Journalism	Osteuropa (Magazine); Editor-in-Chief	Male
128	14.02.2023	13	Gressel, Gustav	Think Tank	Political scientist	European Council on Foreign Relations	Male
129	15.02.2023	16	Masala, Carlo	Academia	Political scientist	Bundeswehr Universität München	Male
130	19.02.2023	394	Heusgen, Christoph	International	International	Munich Security Conference, Chairman	Male
131	20.02.2023	25	Kluge, Janis	Think Tank	Economist	SWP (Stiftung Wissenschaft und Politik)	Male
132	21.02.2023	347	Major, Claudia	Think Tank	Political scientist	SWP (Stiftung Wissenschaft und Politik)	Female
133	22.02.2023	35	Heilmann, Sebastian	Academia	Political scientist	Universität Trier, Professor	Male
134	23.02.2023	21	Stewart, Susan	Think Tank	Political scientist	SWP (Stiftung Wissenschaft und Politik)	Female
135	23.02.2023	13	Kalatschew, Konstantin	Ukraine	Political scientist	Unclear	Male
136	28.02.2023	23	Keupp, Markus	Academia	Economist	ETH Zürich, Militärakademie	Male
137	02.03.2023	303	Masala, Carlo	Academia	Political scientist	Bundeswehr Universität München	Male
138	03.03.2023	31	Clüver Ashbrook, Cathryn	Think Tank	Political scientist	Bertelsmann Stiftung	Female
139	09.03.2023	18	Kosharna, Olga	Ukraine	Consultancy	Independent Expert for Reactor security	Female

TAGESTHEMEN

NO	DATE	LENGTH	NAME	CATEGORY	DISCIPLINE	AFFILIATION
1	12.02.2022	258	Kaim, Markus	Think Tank	Political Scientist	SWP (Stiftung Wissenschaft und Politik);Universität Zürich; Hertie School of Governance
2	14.02.2022	49	Kortunow, Andrej	Think Tank	Historian	Russian International Affairs Council
3	14.02.2022	402	von Fritsch, Rüdiger	International	Ambassador	Ex German Ambassador in RussiaConsultant, Berlin Global Advisors
4	15.02.2022	298	Sasse, Gwendolyn	Academia	Political Scientist	ZOIS (Centre for East European and International Studies); Humboldt- University
5	18.02.2022	418	Hodges, Ben	Military	Military	Ex Commanding general of US Army Europe
6	22.02.2022	28	Chaly, Valeriy	International	Ambassador	Ex Ukrainian Ambassador in the USA
7	22.02.2022	318	Fix, Liana	Think Tank	Political Scientist	Politikwissenschaftlerin, German Marschall Fund
8	22.02.2022	28	Feldmayr, Gabriel	Academia	Economist	Österreichisches Institut für Wirtschaftsforschung
9	24.02.2022	437	Mangott, Gerhard	Academia	Political Scientist	Politikwissenschaftler, Universität Innsbruck
10	25.02.2022	22	Kooths, Stefan	Academia	Economist	Kiel Institut für Weltwirtschaft;
11	25.02.2022	31	Puglierin, Jana	Think Tank	Political Scientist	European Council on Foreign Relations
12	25.02.2022	385	Masala, Carlo	Academia	Political Scientist	Universität der Bundeswehr München
13	27.02.2022	258	Masala, Carlo	Academia	Political Scientist	Universität der Bundeswehr München
14	28.02.2022	315	Kooths, Stefan	Academia	Economist	Kiel Institut für Weltwirtschaft
15	28.02.2022	32	Neitzel, Sönke	Academia	Historian	Historian, Unversität Potsdam
16	28.02.2022	39	Sauer, Frank	Academia	Political Scientist	Politikwissenschaftler, Universität der Bundeswehr München
17	01.03.2022	357	Neitzel, Sönke	Academia	Historian	Historian, Unversität Potsdam
18	04.03.2022	11	Wiegold, Thomas	Journalist	Journalist	Expert Journalist Security
19	04.03.2022	285	Gloger, Katja	Journalist	Journalist	Journalist focus Russia, Board of Journalists without borders
20	04.03.2022	310	Buslavets, Olga	Politician	Politician	Former Minister for Energy, Ukraine
21	06.03.2022	378	Major, Claudia	Think Tank	Political Scientist	SWP (Stiftung Wissenschaft und Politik)
22	07.03.2022	26	Kühn, Ulrich	Academia	Political Scientist	Institut für Friedensforschung und Sicherheitspolitik, Universität Hamburg
23	07.03.2022	17	McManus, Roseanne	Academia	Political Scientist	Pennsylvania State University
24	07.03.2022	18	Pleyer, Severin	Think Tank	Political Scientist	German Institute for Defence and Strategic Studies
25	07.03.2022	425	Gressel, Gustav	Think Tank	Political Scientist	European Council on Foreign Relations
26	08.03.2022	539	Zhovkva, Ihor	Consultancy	Politician	Consultant for foreign affairs of the Ukranien government
27	08.03.2022	16	Zachmann, Georg	Think Tank	Economist	Economist, Thinktank Bruegel
28	09.03.2022	403	Major, Claudia	Think Tank	Political Scientist	SWP (Stiftung Wissenschaft und Politik)
29	09.03.2022	37	Sasse, Gwendolyn	Academia	Political Scientist	ZOIS (Centre for East European and International Studies);University of Oxford
30	10.03.2022	429	Fratzscher, Marcel	Academia	Economist	DIW (Deutsches Institut für Wirtschaftsforschung)Professor for Macroeconomics at Humbold University
31	11.03.2022	20	Meier, Oliver	Academia	Political Scientist	Institute für Friedensforschung und Sicherheitspolitik, Universität Hamburg
32	11.03.2022	524	Münkler, Herfried	Academia	Political Scientist	Humboldt-University (emeritus)
33	12.03.2022	298	Gerald Knaus	Think Tank	Think Tank	European Stability Initiative
34	14.03.2022	332	Zhovkva, Ihor	Consultancy	Politician	Consultant for foreign affairs of the Ukranien government
35	14.03.2022	22	Toews, Thore	Academia	Agroeconomist	Technische Hochschule Bingen
36	14.03.2022	17	Njagi, Timothy	Academia	Agroeconomist	Egerton University Nairobi
37	14.03.2022	20	Husain, Arif	International	Economist	Chiefeconomist World Food Programme
38	15.03.2022	337	Major, Claudia	Think Tank	Political Scientist	SWP (Stiftung Wissenschaft und Politik)
39	15.03.2022	432	Ganijewa, Alissa	Other	Writer	
40	16.03.2022	17	Römmele, Andrea	Academia	Political Scientist	Hertie School of Governance
41	17.03.2022	12	Malet, David	Academia	Political Scientist	American University, Washington D.C.
42	18.03.2022	26	Grigorij, Judin	Academia	Sociologist	Higher School of Economics, Moskow
43	21.03.2022	17	Brückner, Herbert	Academia	Economist	Institut für Arbeitsmarkt- und Berufsforschung; Humboldt-University Berlin
44	22.03.2022	424	Kariakina, Angelina	Journalist	Journalist	Suspilne (Ukrainian TV Channel)
45	23.03.2022	21	Avdevva, Maria	Other	Other	Director, Researchgroup European Expert Association
46	23.03.2022	447	Kurkow, Andrej	Other	Writer	Director, PEN Ukraine
47	23.03.2022	311	Fix, Liana	Think Tank	Political Scientist	Körber Stiftung
48	24.03.2022	31	Schnellenbach, Jan	Academia	Economist	Brandenburgische Technische Universität, Cottbus-Senftenberg
49	26.03.2022	23	Dudek, Antoni	Academia	Historian	Uniwersytet Kardynala Stefana Wyszyńskiego
50	28.03.2022	24	Bayer, Christian	Academia	Economist	Professor for Macroeconomics, Universität Bonn
51	28.03.2022	24	Dullien, Sebastian	Academia	Economist	Institut für Makroökonomie und Konjunkturforschung Düsseldorf; HTW Berlin
52	28.03.2022	522	Birnbaum, Leonhard	Business	Chairman	Chairman E.On (Energy supplier)
53	29.03.2022	14	Struthers, Marie	NGO	NGO	Regional Director Eastern Europe, Amnesty International
54	29.03.2022	17	Peterschmitt, Etienne	International	Agroeconomist	UN Word Food Programme
55	29.03.2022	230	Uhlmannsiek, Janine	NGO	NGO	Amnesty International
56	29.03.2022	14	Mölling, Christian	Think Tank	Political Scientist	Deutsche Gesellschaft für Auswertige Politik
57	30.03.2022	23	Fuest, Clemens	Academia	Legal	ifo Institute
58	30.03.2022	23	Bogner, Matilda	International	International	UN Human Rights Monitoring Mission Ukraine
59	30.03.2022	46	Avdevva, Maria	Other	Other	Director, Researchgroup European Expert Association
60	31.03.2022	31	Krutichin, Michail	Consultancy	Consultant	RusEnergy Consultancy Agency
61	31.03.2022	419	Beck, Marieluise	Think Tank	Think Tank	Zentrum Liberale Moderne
62	02.04.2022	15	Pflugbeil, Sebastian	NGO	Physicist	Ex President, Gesellschaft für Strahlenschutz e.V.
63	03.04.2022	289	Mölling, Christian	Think Tank	Political Scientist	Deutsche Gesellschaft für Auswertige Politik
64	04.04.2022	22	Fücks, Ralf	Think Tank	Think Tank	Zentrum Liberale Moderne
65	05.04.2022	457	Ambos, Kai	Academia	Legal	Professor für Völkerrecht, Universität Göttingen
66	11.04.2022	361	Major, Claudia	Think Tank	Political Scientist	SWP (Stiftung Wissenschaft und Politik)
67	12.04.2022	19	Meier, Oliver	Academia	Political Scientist	Institute für Friedensforschung und Sicherheitspolitik, Universität Hamburg
68	12.04.2022	28	Gressel, Gustav	Think Tank	Political Scientist	European Council on Foreign Relations
69	13.04.2022	30	Fücks, Ralf	Think Tank	Think Tank	Zentrum Liberale Moderne
70	13.04.2022	40	Särkkä, Irro	Academia	Political Scientist	Finish Institute of International Affairs, University of Helsinki
71	13.04.2022	319	Babst, Stefanie	Consultancy	Consultant	Political Consultant, Ex Director Strategic Nato-Planning Group
72	15.04.2022	319	Kropp, Sabine	Academia	Political Scientist	FU Berlin
73	15.04.2022	29	Gontscharow, Stepan	Other	Other	Director, Meinungsforschungsinstitute Lewada-Zentrum
74	19.04.2022	330	Mangott, Gerhard	Academia	Political Scientist	Universität Innsbruck
75	20.04.2022	25	Masala, Carlo	Academia	Political Scientist	Universität der Bundeswehr München
76	20.04.2022	5	Kaim, Markus	Think Tank	Political Scientist	SWP (Stiftung Wissenschaft und Politik);Universität Zürich; Hertie School of Governance
77	21.04.2022	475	Kermani, Navid	Other	Writer	
78	22.04.2022	419	Neitzel, Sönke	Academia	Historian	University of Potsdam
79	26.04.2022	342	Mölling, Christian	Think Tank	Political Scientist	Deutsche Gesellschaft für Auswertige Politik
80	27.04.2022	12	Pepe, Jacopo Maria	Think Tank	Political Scientist	SWP (Stiftung Wissenschaft und Politik)
81	28.04.2022	27	Iwama, Yoko	Academia	Political Scientist	National Graduate Institute for Policy Studies, Japan
82	29.04.2022	335	Strubenhoff, Heinz-Wilhe	Think Tank	Agroeconomist	Brookings Institution
83	02.05.2022	37	Litvinenko, Anna	Academia	Communcation	FU Berlin
84	03.05.2022	23	Kalatschjow, Konstantin	Other	Political Scientist	
85	03.05.2022	270	Kluge, Janis	Think Tank	Economist	SWP (Stiftung Wissenschaft und Politik)
86	04.05.2022	367	Miliband, David	NGO	NGO	President of the International Refugee Committee
87	04.05.2022	23	Nida-Rümelin, Julian	Academia	Political Scientist	LMU München
88	04.05.2022	31	Fücks, Ralf	Think Tank	Think Tank	Zentrum Liberale Moderne
89	04.05.2022	31	Kropp, Sabine	Academia	Political Scientist	FU Berlin
90	04.05.2022	94	Raths, Ralf	Think Tank	Other	Director, Tank Museum Munster
91	06.05.2022	23	Mölling, Christian	Think Tank	Political Scientist	Deutsche Gesellschaft für Auswertige Politik
92	06.05.2022	21	Krajweski, Markus	Academia	Legal	University Erlangen-Nürnberg
93	10.05.2022	316	Urner, Maren	Academia	Neuroscientist	Hochschule HMKW
94	11.05.2022	16	Dullien, Sebastian	Other	Economist	Institut für Makroökonomie und Konjunkturforschung Düsseldorf
95	12.05.2022	37	Umland, Andreas	Academia	Political Scientist	Stockholm Centre for Eastern European Studies
96	19.05.2022	344	Fischer, Sabine	Think Tank	Political Scientist	SWP (Stiftung Wissenschaft und Politik)

97	25.05.2022	25	Fjordorow, Jurij	Other	Other	N/A
98	25.05.2022	333	Wiegold, Thomas	Journalist	Journalist	Freelance
99	26.05.2022	14	Klett, Tomas Casas	Academia	Economist	University St. Gallen
100	31.05.2022	278	Kempfert, Claudia	Academia	Economist	Deutsches Institut für Wirtschaftsforschung (DIW); Leuphana University Lüneburg
101	07.06.2022	136	Conrad, Gerhard	Other	Other	Ex BND Agent
102	08.06.2022	18	Krotofil, Marina	Other	Other	European Network of Cybersecurity
103	08.06.2022	15	Atung, Manuel	NGO	NGO	Arbeitsgruppe Kritische Infrastruktur
104	09.06.2022	459	Gressel, Gustav	Think Tank	Political Scientist	European Council on Foreign Relations
105	14.06.2022	403	Knaus, Gerald	Think Tank	Think Tank	European Stability Initiative
106	17.06.2022	57	Martschuk, Anton	NGO	Other	Anti-Corruption Action Centre
107	17.06.2022	29	Fücks, Ralf	Think Tank	Think Tank	Zentrum Liberale Moderne
108	17.06.2022	30	Subarewitsch, Natalja	Academia	Geography	Lomonossow University Moscow
109	20.06.2022	16	Schabas, William	Academia	Legal	University of Middlesex; Leiden University
110	23.06.2022	28	Haran, Oleksij	Academia	Political Scientist	National University of Kyiv-Mohyla
111	22.07.2022	12	Schnitzer, Monika	Academia	Economist	LMU München
112	25.07.2022	15	Kempfert, Claudia	Academia	Economist	Deutsches Institut für Wirtschaftsforschung (DIW)
113	29.07.2022	223	Brakel, Christian	Other	Other	Director Heinrich-Böll-Stiftung, Istanbul
114	30.07.2022	18	Bareli, Avi	Academia	Historian	Ben Gurion University, Negev
115	02.08.2022	14	Matwijtschuk, Oleksandr	Other	Legal	Centre for Civil Liberties
116	04.08.2022	30	Lamerty, Pia	Other	Psychologist	Director, Centre für Monitoring, Analyse und Strategie
117	05.08.2022	10	Fuest, Clemens	Academia	Economist	ifo Institute
118	05.08.2022	15	Bach, Stefan	Academia	Economist	Deutsches Institut für Wirtschaftsforschung (DIW)
119	05.08.2022	14	Adam, Stuart	Think Tank	Economist	Institute for Fiscal Studies
120	05.08.2022	40	Sopova, Alisa	Academia	Anthropologist	Princeton University
121	08.08.2022	17	Krebs, Tom	Academia	Economist	University of Mannheim
122	08.08.2022	16	Schularick, Moritz	Academia	Economist	University of Bonn
123	10.08.2022	25	Neitzel, Sönke	Academia	Historian	University of Potsdam
124	15.08.2022	18	Satpajew, Dosym	Other	Political Scientist	Freelance
125	16.08.2022	25	Mölling, Christian	Think Tank	Political Scientist	Deutsche Gesellschaft für Auswertige Politik
126	25.08.2022	16	Grossi, Rafael	International	International	Director, International Atomic Energy Association (IAEA)
127	25.08.2022	20	Meister, Stefan	Think Tank	Political Scientist	Deutsche Gesellschaft für Auswertige Politik
128	29.08.2022	281	Hirth, Lion	Academia	Economist	Hertie School of Governance
129	01.09.2022	26	Grossi, Rafael	International	International	Director, International Atomic Energy Association (IAEA)
130	01.09.2022	33	Mölling, Christian	Think Tank	Political Scientist	Deutsche Gesellschaft für Auswertige Politik
131	02.09.2022	53	Grossi, Rafael	International	International	Director, International Atomic Energy Association (IAEA)
132	02.09.2022	290	Smital, Heinz	NGO	Physicist	Greenpeace
133	05.09.2022	15	Kempfert, Claudia	Think Tank	Economist	Deutsches Institut für Wirtschaftsforschung (DIW)
134	10.09.2022	10	Richter, Wolfgang	Think Tank	Military	SWP (Stiftung Wissenschaft und Politik)
135	12.09.2022	20	Ischinger, Wolfgang	International	International	Ex Director Münchner Sicherheitskonferenz
136	23.09.2022	18	Kyrytschenko, Julia	Think Tank	Think Tank	Center of Policy and Legal Reform
137	27.09.2022	353	Klein, Margarete	Think Tank	Political Scientist	SWP (Stiftung Wissenschaft und Politik)
138	28.09.2022	28	Peters, Johannes	Academia	Political Scientist	Institut für Sicherheitstechnik, Kiel
139	28.09.2022	356	Neumann, Peter R.	Academia	Political Scientist	King's College London
140	30.09.2022	422	Glukhovsky, Dmitry	Other	Writer	Freelance
141	08.10.2022	14	Kozii, Ihor	Think Tank	Think Tank	Institut für Euro-Atlantische Zusammenarbeit, Kiew
142	12.10.2022	27	Maraharens, Sören	Military	Military	Kompetenzzentrum gegen Hybride Bedrohung
143	12.10.2022	21	Meister, Stefan	Think Tank	Political Scientist	Deutsche Gesellschaft für Auswertige Politik
144	13.10.2022	351	Plagemann, Johannes	Academia	Political Scientist	Leibniz Institut, Hessische Stiftung Friedens- und Konfliktforschung
145	19.10.2022	18	Jakovenko, Valerij	Other	Other	Freelance
146	21.10.2022	353	Zhadan, Serhij	Other	Writer	Freelance
147	21.10.2022	14	Starski, Paulina	Academia	Legal	University of Freiburg
148	24.10.2022	26	Betlij, Oleksandra	Think Tank	Economist	Institute for Economic Research and Policy Consuling, Kiew
149	12.11.2022	18	Mölling, Christian	Think Tank	Political Scientist	Deutsche Gesellschaft für Auswertige Politik
150	13.11.2022	15	Alt, Volker	Other	Doctor	University of Regensburg
151	15.11.2022	44	Gallijamow, Abbas	Think Tank	Political Scientist	Foreing Policy Research Institute
152	15.11.2022	298	Pagung, Sarah	Think Tank	Political Scientist	Deutsche Gesellschaft für Auswertige Politik
153	16.11.2022	16	Gressel, Gustav	Think Tank	Political Scientist	European Council on Foreign Relations
154	20.11.2022	14	Satpajew, Dosym	Other	Political Scientist	Freelance
155	29.11.2022	27	Vyshlinsky, Hlib	Think Tank	Think Tank	Centre for Economic Strategy, Ukraine
156	01.12.2022	14	Wolff, Alan	International	International	Ex General Director, World Trade Organisation
157	05.12.2022	301	Wüstner, André	Military	Military	Deutscher Bundeswehrverband, Director
158	13.12.2022	331	Mangott, Gerhard	Academia	Political Scientist	Universität Innsbruck
159	21.12.2022	290	Mölling, Christian	Think Tank	Political Scientist	Deutsche Gesellschaft für Auswertige Politik
160	27.12.2022	26	Schelest, Hannah	Think Tank	Political Scientist	Ukrainian Prism, Foreign Policy Council
161	05.01.2023	39	Puglierin, Jana	Think Tank	Political Scientist	European Council on Foreign Relations
162	06.01.2023	380	Major, Claudia	Think Tank	Political Scientist	SWP (Stiftung Wissenschaft und Politik)
163	11.01.2023	14	Selesnyov, Vladyslav	Other	Other	
164	12.01.2023	17	Deitelhoff, Nicole	Academia	Political Scientist	Leibniz Institut, Hessische Stiftung Friedens- und Konfliktforschung
165	12.01.2023	26	Schipper, Sebastian	Academia	Geographer	Goethe-University of Frankfurt
166	17.01.2023	17	Franke, Ulrike	Think Tank	Political Scientist	European Council on Foreign Relations
167	17.01.2023	21	Wüstner, André	Military	Military	Deutscher Bundeswehrverband, Director
168	20.01.2023	420	Hodges, Ben	Military	Military	Ex Commanding general of US Army Europe
169	21.01.2023	16	Hellestveit, Cecillie	Academia	Legal	Institute for Human Rights, Norway
170	22.01.2023	11	Besch, Sophia	Think Tank	Political Scientist	Carnegie Endowment for International Peace
171	25.01.2023	17	Börzel, Tanja	Academia	Political Scientist	FU Berlin
172	25.01.2023	19	Wüstner, André	Military	Military	Deutscher Bundeswehrverband, Director
173	25.01.2023	17	Masala, Carlo	Academia	Political Scientist	Universität der Bundeswehr München
174	25.01.2023	322	Deitelhoff, Nicole	Academia	Political Scientist	Leibniz Institut, Hessische Stiftung Friedens- und Konfliktforschung
175	31.01.2023	25	Stuenkel, Oliver	Other	Political Scientist	Getulio Vargas Foundation
176	31.01.2023	347	von Fritsch, Rüdiger	Consultancy	Consultant	Ex German Ambassador in Moskau
177	02.02.2023	7	Nikolov, Yuri	Journalist	Journalist	Investigative Journalism ZN.UA
178	02.02.2023	20	Ryzhenko, Kateryna	NGO	NGO	Transparency International Ukraine
179	03.02.2023	15	Mölling, Christian	Think Tank	Political Scientist	Deutsche Gesellschaft für Auswertige Politik
180	04.02.2023	26	Zachmann, Georg	Think Tank	Economist	Think tank Bruegel
181	08.02.2023	14	Crump, Justine	Business	Consultant	CEO, Sibylline Ltd, Advisory firm global security strategies
182	14.02.2023	17	Mölling, Christian	Think Tank	Political Scientist	Deutsche Gesellschaft für Auswertige Politik
183	14.02.2023	14	Wiegold, Thomas	Journalist	Journalist	Freelance
184	18.02.2023	266	Ischinger, Wolfgang	International	International	Ex Director Münchner Sicherheitskonferenz
185	19.02.2023	13	Havryshko, Marta	Academia	Historian	University of Basel
186	21.02.2023	333	Pagung, Sarah	NGO	Political Scientist	Körber Stiftung
187	22.02.2023	12	Filatova, Irina	Academia	Historian	National Research University Higher School of Economics, Moskow
188	24.02.2023	23	Betlij, Oleksandra	Think Tank	Economist	Institute for Economic Research and Policy Consuling, Kiew
189	24.02.2023	229	Romanenko, Darya	NGO	NGO	Help - Hilfe zur Selbsthilfe e.V.
190	24.02.2023	331	Schlögel, Karl	Academia	Historian	University of Konstanz (emeritus)
191	25.02.2023	16	Nachtwey, Oliver	Academia	Sociologist	Philosopisch-Historische Fakultät, University of Basel
192	27.02.2023	15	Mölling, Christian	Think Tank	Political Scientist	Deutsche Gesellschaft für Auswertige Politik
193	03.03.2023	12	Stelzenmüller, Constanz	Think Tank	Political Scientist	Brookings Institution
194	03.03.2023	18	Rathke, Jeffrey	Academia	International	John Hopkins University, American Institute for Contemporary German Studies
195	06.03.2023	22	Römmele, Andrea	Academia	Political Scientist	Hertie School of Governance

196	07.03.2023	285	Mölling, Christian	Think Tank	Political Scientist	Deutsche Gesellschaft für Auswertige Politik

Appendix 4: Coding of Questions Experts Were Asked

OBJECTS OF QUESTIONS	HEUTE JOURNAL	TAGESTHEMEN
What are the lessons of history?	I (1)	IIIII (5)
How does this look from Putin's perspective?	IIIIIIIIIIIIIIIIIIIIIIII (24)	IIIIIIIIIIIIIII (15)
What could security dialogue look like?	IIIII (5)	III (3)
What does the future look like?	IIIIIIIIIIIIIIIIII (18)	IIIIIIIIIIIIIIIIIIIIIIIIIII (27)
What is happening?	IIIIIIIIIIIIII (15)	IIIIIIIIIIIIIII (15)
Is this a genocide/ does Russia commit war crimes?	I (1)	I (1)
Is this measure a good idea?	IIIIIIIII (11)	IIIIIIIIIII (11)
What should we do now?	(0)	III (3)
How is May 9 remembered in Russia?	I (1)	(0)
What is German government/ NATO/ USA thinking?	IIIIII (6)	IIIIIII (7)
Can we take Schröder's initiative for peace seriously?	I (1)	(0)
What is Ukrainian government thinking?	I (1)	(0)
Is it common that we know so little about numbers of deaths among warring troops?	(0)	I (1)
How is the war seen in Asia/Global South/China?	(0)	III (3)
What is the German population thinking?	(0)	II (2)
